# Small Hearts, Big Clues: A Narrative Review on Sex-Related Disparities in the Diagnosis and Management of Cardiac Amyloidosis in Women

**DOI:** 10.3390/jcm15124819

**Published:** 2026-06-21

**Authors:** Ilenia Monaco, Mounia Sedrati, Insaf Chouarfia, Fatima Zahra Samet Bouhaik, Valeria Trivelloni, Yassine Bencharef, Mohammed Fouad Sekkal, Dario Bottigliero

**Affiliations:** Department of Cardiology, Centre Hospitalier General Victor Jousselin de Dreux, 28100 Dreux, France

**Keywords:** amyloidosis, transthyretin, cardiac amyloidosis, women, sex differences, ATTR, diagnostic delay, gender disparities, heart failure

## Abstract

**Background:** Amyloidosis is an infiltrative cardiomyopathy caused by amyloid deposition into the myocardium. In recent years, recognition of this treatable cause of heart failure has increased. There are striking sex differences in the diagnosis, clinical course and outcome of the disease. Notably, women have a worse prognosis than men with similar amounts of cardiac involvement. **Methods:** This review provides an overview of the current state of knowledge regarding the epidemiology, clinical features, diagnosis and treatment of amyloid heart disease. The differences observed between men and women are discussed, and recent advances in the field are highlighted. **Results:** Compared to men, women are generally older at diagnosis, appear to have less severe cardiac disease at the time of impairment and are more frequently diagnosed late. The less apparent disease manifestations in women may be responsible for the delay in diagnosis. Moreover, women may be underdiagnosed when sex-neutral diagnostic criteria are used. **Conclusions:** Addressing diagnostic disparities may require the use of sex-specific diagnostic thresholds, as well as a more expansive use of multimodality imaging. Future clinical trials should aim to enroll a greater number of female participants to inform optimal therapeutic approaches and to define the sex-specific disease phenotype for this increasingly treatable disease.

## 1. Introduction

### 1.1. Disease Definition and Clinical Significance

Amyloidosis is a condition affecting the whole body in which amyloid protein accumulates in tissues and organs. This condition tends to have a poor prognosis when it affects the heart. Clinically, it can mimic left ventricular hypertrophy of other etiologies and so can be notoriously difficult to diagnose [1,2,3,4,5]. There are two main forms of cardiac amyloidosis: transthyretin (ATTR) amyloidosis and light-chain (AL) amyloidosis. ATTR may or may not have a genetic cause. The majority of cases of hereditary transthyretin amyloidosis (hATTR) are caused by mutations in the transthyretin gene that result in the production of a variant transthyretin protein. Wild-type transthyretin amyloidosis (wtATTR) accounts for the majority of cases of transthyretin amyloidosis [6]. While the diagnosis of cardiac amyloidosis has traditionally been based on clinical features supported by biopsy evidence of amyloid, the diagnosis can now be established without biopsy in many cases using imaging techniques. These include technetium-99m pyrophosphate scintigraphy and cardiovascular magnetic resonance imaging, particularly native T1 mapping [7].

Cardiac amyloidosis can clinically be mistaken for Hypertrophic Cardiomyopathy in patients with left ventricular hypertrophy. Among cardiomyopathies with a hypertrophic phenotype, non-sarcomeric phenocopies—including amyloidosis, Fabry disease, glycogen storage disorders, RASopathies, and mitochondrial diseases—account for a substantial proportion of cases (about 11%) of all forms of cardiomyopathies. Because effective, disease-specific treatments are now available, a differential diagnosis is crucial for the patients’ prognosis [8].

### 1.2. Biomolecular Mechanisms and Pathogenic Underpinnings

Cardiac amyloidosis is a progressive condition in which the heart muscle is infiltrated by protein aggregates called amyloid fibrils. Two types of amyloid proteins usually cause this disease: wild-type transthyretin (ATTR) and the light chains of immunoglobulins (AL). The process starts with the destabilization and subsequent dissociation of the native, functional TTR tetramer or the light chain from its heavy chain partner. The resulting soluble oligomers are highly proteotoxic to the cells. They can penetrate the cells and cause damage to the mitochondria (leading to a selective decrease in the activity of the oxidative phosphorylation complexes), disrupt the handling of calcium ions, and trigger cell death programs (apoptosis) in various other ways. The resulting amyloid fibrils are very stable and accumulate in the extracellular space of the myocardium. Over time, this can cause a thickening of the myocardial walls, leading to a so-called restrictive cardiomyopathy. Although the deposits of amyloid proteins in the myocardium themselves cause ongoing damage to the heart, a so-called pathophysiologic effect of amyloid deposits in the myocardium, the amount of deposits and the pathophysiologic effect are not necessarily correlated, and the mechanisms of this effect are not yet fully understood [9].

### 1.3. Sex-Based Epidemiological Shifts and Diagnostic Recognition

The epidemiology of amyloidosis has recently changed due to FDA approval of the first disease-modifying therapies for hereditary transthyretin amyloidosis (hATTR), increasing awareness of the disease and screening of high-risk populations. Remarkably, female patients represent an increasing proportion of amyloidosis patients, which is in contrast to the previously reported male preponderance [4].

In a single-center cohort of 140 consecutive patients with a symptomatic diagnosis of wild-type (wtATTR) or hereditary transthyretin amyloidosis (hATTR), 11.4% of the patients before 2019 were women. In contrast, 16.7% (10/60) of the patients with ATTR amyloidosis in 2019–2022 were women. This increase in the proportion of women with ATTR amyloidosis reflects an improvement in screening [10].

Active screening strategies involving 99mTc-pyrophosphate SPECT/CT in a diverse population of patients with heart failure and left ventricular hypertrophy for suspected or documented cardiac amyloidosis identified that 31.3% of the screened patients were women. Compared to referral-based cohorts, this proportion was significantly increased (13.3%, *p* < 0.001) [11]. In a screening cohort of 344 patients with confirmed or suspected transthyretin amyloidosis seen at the Amyloid Center at the Massachusetts General Hospital, women comprised only 4.1% of those <70 years but 14.3% of those >90 years [2].

A prospective German survey of 118 patients with amyloidosis diagnosed between 2020 and 2021 revealed that 30% of the study group were women with a median diagnostic delay of 9.0 months. Overall, there has been a rising number recognized amyloidosis cases in women in recent years, and better treatment is now available [12].

### 1.4. Diagnostic Delays: Magnitude and Recent Improvements

Early diagnosis is essential in the management of amyloidosis. The median diagnostic delay in 34 patients with wtATTR who were <70 years old was 5.2 years (range 1.2–22 years) in women and 1.3 years (range 0.3–7.4 years) in men (*p* < 0.001). The median diagnostic delay in a Danish cohort of 50 patients with wtATTR in the period 2017–2019 was 13 months, with considerable variations among individual patients, with a range of 2 to 47 months [12,13]. The diagnostic delay was associated with more severe symptoms and a more impaired left ventricular diastolic dysfunction at the time of diagnosis. However, the diagnostic processes are rapidly evolving. A single-center temporal analysis of the time-to-diagnosis in women with cardiac symptoms showed a dramatic reduction in time-to-diagnosis in recent years. The median time-to-diagnosis decreased from a median of 750 days (range 128–1058 days) in the period June 2018/2021 to 86 days (range 23–284 days) in the period 07/2021–2024, corresponding to an 8.7-fold reduction in the time to diagnosis for women with cardiac symptoms. Improved awareness of the disease and the implementation of screening protocols are most likely responsible for these findings [1,14].

## 2. Methods

### 2.1. Database Search and Study Selection

We conducted a targeted systematic review of the literature on amyloidosis with emphasis on sex and gender differences, cardiac manifestations, and systemic involvement, following Preferred Reporting Items for Systematic Reviews and Meta-Analyses (PRISMA) guidelines. Using PubMed (MEDLINE), Scopus, and Web of Science databases, we combined MeSH controlled vocabulary terms including “Amyloidosis/diagnosis”, “Amyloidosis/epidemiology”, “Amyloidosis/therapy”, “Amyloidosis/pathology”, “Heart Diseases/diagnosis”, “Cardiomyopathy/diagnosis”, “Transthyretin/genetics”, “Sex Factors”, and “Sex-Based Differences in Disease and Health” with relevant free-text keywords including “amyloidosis women”, “amyloidosis female”, “cardiac amyloidosis”, “cardiac ATTR”, “transthyretin amyloidosis”, “light chain amyloidosis”, “AL amyloidosis”, “sex differences amyloidosis”, “gender disparities amyloidosis”, “female amyloidosis phenotype”, and “sex-specific diagnosis”. The search was expanded to include publications between 1 January 2020, and 28 February 2026, to capture both recent evidence and foundational studies from the past two decades, reflecting evolving diagnostic and therapeutic approaches. An additional hand search of reference lists from included studies and relevant review articles was conducted to identify potentially missed citations.

### 2.2. Inclusion and Exclusion Criteria

Two independent reviewers systematically screened all retrieved citations using standardized screening forms. The initial search strategy retrieved 1247 unique articles from combined database searches across the 2020–2026 timeframe. After removal of 89 duplicate entries identified by both automated deduplication tools and a manual review, 1158 articles underwent title and abstract screening. During this phase, 743 articles were excluded based on clear non-relevance. The remaining 415 full-text articles were retrieved and evaluated in detail by both reviewers using a standardized assessment tool. Disagreements regarding study inclusion were resolved through consensus discussion.

Inclusion criteria were: (1) original peer-reviewed studies including randomized controlled trials, prospective observational cohort studies, retrospective cohort studies, case–control studies, or case series published 2020–2026; (2) systematic reviews and meta-analyses addressing amyloidosis; (3) studies published in English; (4) studies reporting clinical, diagnostic, epidemiological, pathological, or therapeutic data regarding amyloidosis; (5) studies that either explicitly provided sex-stratified analyses or enrolled sufficient female patients to allow gender-based comparisons; (6) studies including patients with a histopathologically confirmed amyloidosis diagnosis or radiologically diagnosed amyloidosis meeting established diagnostic criteria; and (7) studies reporting outcomes including diagnostic accuracy, clinical manifestations, treatment response, survival, or complications.

Exclusion criteria were: (1) case reports limited to single patients; (2) conference abstracts or editorials without primary data; (3) non-English language publications; (4) studies with exclusively pediatric populations (age < 18 years); (5) studies lacking sex-stratified data or explicitly addressing sex-based differences; (6) studies focused solely on hereditary transthyretin amyloidosis variants without cardiac involvement; and (7) animal studies or in vitro research.

**Figure 1:**
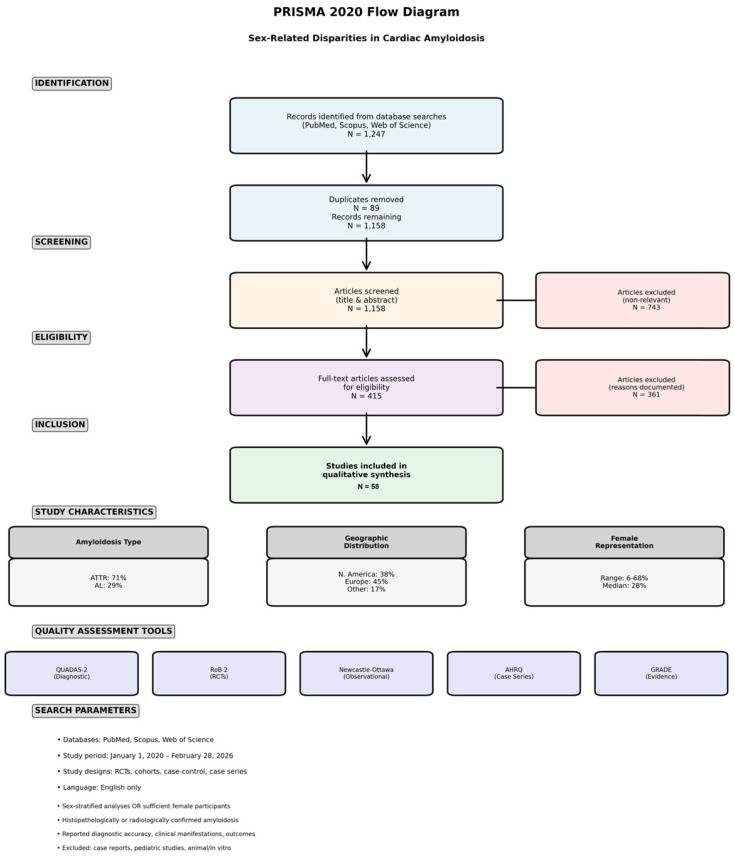
PRISMA flow diagram illustrating the study selection process, including the number of records identified, screened, assessed for eligibility, and included in the final analysis.

Fifty-eight studies ultimately met all inclusion criteria and were included in the qualitative synthesis. Among the included studies, 71% focused specifically on transthyretin (ATTR) amyloidosis, including both wild-type and hereditary; 29% examined light-chain (AL) amyloidosis or included comparative analyses. Patient age at study enrollment ranged from 18 to 95 years, with most ATTR amyloidosis cohorts focused on patients aged > 65 years, while AL amyloidosis cohorts frequently included younger patients. The proportion of women in the included studies ranged from 6% to 68%, with a median of 28% female representation across all studies. Geographic representation included studies from North America (38%), Europe (45%), and other regions (17%) (Figure 1).

## 3. Discussion

### 3.1. Clinical Presentation and Sex-Specific Phenotypes

#### 3.1.1. ATTR Phenotypic Variations in Women

Differences exist in the clinical presentation of amyloidosis between women and men. In a recently established Spanish registry of 385 patients with a definite or suspected diagnosis of transthyretin (ATTR) amyloidosis, including 49 patients with amyloid light-chain (AL) amyloidosis, women with late-stage symptoms of severe heart failure were overrepresented (36.8% of women vs. 25.2% of men, *p* = 0.031) [15]. Cardiac amyloidosis can be associated with significant conduction system disorders in a large portion of patients, with significant conduction disease requiring a pacemaker in up to 40% of them. Most of the recent information regarding conduction abnormalities in transthyretin cardiac amyloidosis has demonstrated marked sex disparities in the prevalence and presentation of conduction abnormalities in this subgroup of amyloidosis [8]. For example, in a geriatric population with arrhythmias and conduction disturbances, the prevalence of conduction abnormalities is 1.8-fold greater in men than in women, with the following distribution: first-degree AV block (35% vs. 20%, *p* = 0.007) and left bundle-branch block (29% vs. 13%, *p* = 0.001) being the most common findings of conduction abnormalities in this population [16]. The prevalence of atrial fibrillation and/or flutter in a consecutive series of 94 patients with primary left ventricular dysfunction secondary to transthyretin CM (ATTR-CM) seen at our center was 66%, with 66.5% of the patients being diagnosed by symptoms or monitoring, and 29.0% incidentally found during follow-up or device interrogation [17,18].

#### 3.1.2. AL Amyloidosis Epidemiology in Women

Female patients have a preponderance of AL amyloidosis in comparison to the gender-matched prevalence of patients with ATTR amyloidosis. An expert center-based study identified AL amyloidosis in 57.1% of 63 patients with amyloid cardiomyopathy. Females comprised 39.7% of the AL group [19]. A clustering-based phenotypic study showed a higher prevalence of AL amyloidosis in women who presented early and had multiorgan involvement [20]. A retrospective analysis of 28 patients with heavy-chain amyloidosis and seven with heavy- and light- chain amyloidosis found that 68% of the patients with AH amyloidosis were females (19/28) and 32% were males (9/28). The mean age at diagnosis was 73 years for both heavy-chain groups [21]. A single-center prospective German study of 118 patients with amyloidosis diagnosed between 2020 and 2021 found that 30% of the patients were females with a median time to diagnosis of 9.0 months [12].

### 3.2. Sex-Specific Cardiac Structural Phenotype in Amyloidosis

#### 3.2.1. Left Ventricular Wall Thickness and Indexed Parameters

Involvement of cardiac amyloidosis differs between the sexes. Left ventricular (LV) wall thicknesses are a case in point [22]. In a German cohort study of 240 patients with AL- and ATTR-amyloidosis, the involvement was significantly less in women compared to men. The study group consisted of 34 women (14.2%) and included 7.7% of the patients in the male-dominated cohort. Women had a thinner interventricular septum (15.9 ± 4.5 mm vs. 18.1 ± 4.5 mm, *p* = 0.026) and lower stroke volumes (49 vs. 55 mL, *p* = 0.022) [1]. Larger studies from Italian cardiac amyloidosis centers, including 302 patients with varying forms of cardiac amyloidosis, included 62 women (20%) in the study group. Women had significantly lower interventricular septal thickness (15 mm vs. 17 mm, *p* < 0.001) and lower posterior wall thickness (13 mm vs. 15 mm, *p* < 0.001). When thicknesses were indexed for body surface area, the sex-specific differences disappeared. Apparently, the differences are based on the different physiological cardiac sizes of the sexes. However, a seemingly normal left ventricular wall thickness (≤1.2 cm) was observed in 13% of patients with AL amyloidosis and 6.5% of patients with ATTR amyloidosis. Notably, 68% of these patients showed an elevated relative wall thickness (RWT > 0.42). Indexing wall thickness for body surface area therefore also allows a more sensitive diagnostic approach in women [22,23].

#### 3.2.2. Ejection Fraction Paradoxes and Functional Impairment

Despite clinical evidence of cardiac disease, women with cardiac manifestations of transthyretin amyloidosis show a paradoxically normal or elevated left ventricular ejection fraction (LVEF) [24]. In the largest single-center cohort of 1062 wild-type patients at the French Cardiac Amyloidosis Reference Center, women with wtATTR amyloidosis showed a higher LVEF of 52% as compared to men (50%; *p* < 0.05) [2]. Similar results were found in the Spanish national registry of 385 patients with hereditary and wild-type ATTR amyloidosis (56.0% vs. 52.6% in men; *p* = 0.003) [15]. A single-center study from Germany included 75 women with wild-type ATTR amyloidosis and also found a higher LVEF of 55% as compared to men (51.7%; *p* < 0.001). However, in contrast to men, women with cardiac transthyretin amyloidosis have worse symptoms despite preserved LVEF [1]. In the Spanish registry, women had more severe heart failure symptoms (advanced NYHA class III-IV in 36% as compared to 25% of men; *p* = 0.04). This clinical observation has not been fully elucidated. It has been hypothesized that sex-specific patterns in gene expression or distinct biochemical interactions between amyloid fibrils and myocardial cells might modulate this phenotype, representing an important area for future mechanistic investigation [15].

While conventional echocardiography and CMR are unable to quantify the degree of functional impairment in cardiac amyloidosis, cardiopulmonary exercise testing (CPET) allows for the assessment of cardiorespiratory performance and thereby provides an independent prognostic parameter for clinical decision-making. Importantly, CPET is sex-neutral and allows for an objective assessment of functional capacity, which is superior to the assessment by NYHA functional class. Women with a preserved LVEF and ATTR have been found to suffer from functional limitation of unknown pathophysiology, referred to as the functional limitation paradox. Recent studies have identified that women with a preserved LVEF and ATTR-CM have a significant impairment in peak VO_2_ as measured by CPET while measurements of left ventricular ejection fraction by imaging studies are preserved. Moreover, in a large study of over 1400 outpatients with heart failure with a normal or only mildly impaired 6MWD, peak VO_2_ measured during CPET provided superior risk stratification for mortality. Notably, there are important sex-specific differences in the pathophysiologic mechanisms that lead to decreased exercise capacity in patients with heart failure with a preserved ejection fraction. While women with HFpEF are characterized by a decreased exercise capacity due to stiff left ventricles and attenuated peripheral oxygen extraction, men are characterized by a decreased exercise capacity due to biventricular dilatation and a failure to increase stroke volume. By including these parameters into the biomarker staging of cardiac amyloidosis and risk stratification, using a combination of biomarkers and imaging studies, it is now possible to accurately assess clinical risk in women with the preserved-LVEF form of the disease and to individualize treatment during disease-modifying therapy [25,26,27,28].

Women demonstrate intrinsic physiological differences in left ventricular structure and contractile function that fundamentally shape how cardiac disease manifests clinically. Women preferentially exhibit smaller LV cavity dimensions and lower LV end-systolic volumes—geometric features that, paradoxically, support the maintenance of the ejection fraction through enhanced contractility rather than reflecting superior myocardial health.

LV end-systolic elastance, a contractile, afterload-independent parameter, has been shown to be increased in healthy women because of their small left ventricular chamber size.

During aging, this sex-specific pattern persists: women consistently demonstrate smaller ventricular dimensions and a lower LV mass compared to men, yet exhibit higher biventricular systolic function. It is this unique pattern of sex-specific small ventricles with high contractile performance that becomes a limitation in the setting of infiltrative cardiac disease, such as cardiac amyloidosis, where the acutely stiff myocardium is further compromised by the geometric constraints of the female heart, resulting in the clinical preserved-LVEF phenotype of cardiac failure [29].

#### 3.2.3. Systemic Features and Extracardiac Manifestations

Several extracardiac manifestations of amyloidosis are often first recognized in women and predate the diagnosis of cardiac amyloidosis. In Val122Ile ATTR amyloidosis, women had a higher prevalence of neuropathy (69% vs. 56%, *p* = 0.002) and CTS (38% vs. 27%, *p* = 0.03) among 513 patients. Prior CTS was a strong predictor of cardiac amyloidosis [30].

Among 108 patients with amyloidosis, 36% had a prior CTS diagnosis, and those with a prior CTS more frequently presented with subsequent cardiac amyloidosis (78% vs. 53%, *p* = 0.013) [31].

Among 752 with hereditary ATTR amyloidosis patients in the REACT registry, women presented with the disease at a younger age but had a longer tome to diagnosis. A mixed phenotype presentation (both cardiac and neuropathic involvement) was also more common in women (42.3% vs. 28.8%) [32].

Women with systemic amyloidosis present with symptoms of neuropathy or systemic manifestations prior to cardiac symptoms. Among 513 patients with Val122Ile associated amyloidosis, 61% were women. Women (69%) more commonly had neuropathy than did men (56%; *p* = 0.002). Carpal tunnel syndrome (Cts) occurred in 38% of women and 27% of men (*p* = 0.03) [30]. Prior Cts was the most significant predictor of subsequent cardiac amyloidosis, with a mean delay of 4 years prior to the diagnosis of amyloidosis [31]. In a consecutive series of 98 patients undergoing carpal tunnel release surgery, 10.2% of the tenosynovium biopsies had amyloid deposition [33]. In the REACT registry, women with hATTR presented at a younger age (mean 49 vs. 55 years; *p* < 0.0001) but had a longer time to diagnosis (mean 6.5 vs. 4.5 years; *p* < 0.0001). Notably, 42.3% of the women presented with a mixed phenotype (both cardiac and neuropathic) [32].

### 3.3. Sex-Specific Diagnostic Challenges and Thresholds

#### 3.3.1. Wall Thickness Diagnostic Criteria and Systematic Bias

Suspecting cardiac amyloidosis should be considered when the left ventricular wall thickness is ≥ 12 mm in an appropriate clinical context. However, women with ATTR have thinner septal walls, and the currently used universal thickness threshold for the initial suspicion of amyloidosis may fail to account for important sex-specific differences in cardiac dimensions and is likely to introduce a diagnostic delay in women [1,23].

To reduce systematic diagnostic delays, future research should prospectively test the hypothesis that adapting screening thresholds to >11 mm for female patients improves diagnostic accuracy without exponentially increasing false-positive rates [23].

Sex-specific cut-points for left ventricular wall thickness are increasingly recognized in daily practice. However, the current evidence consists mainly of observational studies and abstracts that are limited by small numbers of patients, single-center designs, and retrospective data collection. Therefore, before these newly proposed cut-points can be translated into routine clinical practice, prospective multicenter validation studies are warranted to determine whether a lower threshold for the diagnosis of cardiac amyloidosis in women will improve the accuracy of the diagnosis and not be associated with a marked increase in false-positive screening tests and subsequent testing, including bone scintigraphy and a cardiac biopsy. In the interim, these cut-points should be used as a clinical reminder of decreased LV wall thickness in women. Furthermore, other echocardiographic features that are characteristic of CA and are present in women with borderline LV wall thickness, such as increased relative wall thickness, apical sparing, and reduced longitudinal strain, should be used in the differential diagnosis [34].

#### 3.3.2. Echocardiographic Diagnostic Accuracy and Performance

The diagnostic performance of different echocardiographic parameters for left ventricular diastolic dysfunction varies. A systematic review and meta-analysis of 35 studies that evaluated the accuracy of echocardiography for left ventricular diastolic dysfunction were performed. The best traditional parameter varied slightly between the sexes and had a sensitivity of 72% at a fixed 95% specificity, and the second-best novel parameter had a sensitivity of 64% at a fixed 95% specificity. However, the sensitivity of the longitudinal strain parameters (global longitudinal strain and endocardial fractional shortening) was the highest at 78%. There was greater variability among studies for specificity, overall accuracy, and predictive values compared with sensitivity and specificity. Data were not available for a meta-analysis of sex-specific performance [35,36].

In a prospective study of 75 patients with cardiac amyloidosis confirmed by cardiac biopsy, echocardiography was useful for diagnosing cardiac amyloidosis and for characterizing the amyloid cardiomyopathy caused by amyloid light-chain (AL) versus transthyretin (ATTR). Echocardiographic indicators of wall thickness and diastolic dysfunction were increased ≥ 7 years before the clinical diagnosis of amyloidosis. The relative wall thickness (RWT) was increased in patients with ATTR (0.74 ± 0.15 vs. 0.62 ± 0.10, *p* = 0.004), whereas the LV mass index (144 ± 40 vs. 115 ± 29 g/m^2^, *p* = 0.020) and LVEF (50% ± 10% vs. 60% ± 9%, *p* = 0.009) were increased in patients with ATTR versus AL amyloidosis [37].

Left ventricular ejection fraction (LVEF) and other conventional echocardiographic parameters cannot accurately reflect the degree of cardiac dysfunction in patients with cardiac amyloidosis, in particular in women presenting with a preserved systolic function but severe exercise intolerance and poor clinical outcomes. On the other hand, an assessment of myocardial work, which reflects the integration between systolic and diastolic function and also the efficiency of increased pressure generation in relation to increased deformation of the myocardium during work, as determined by pressure–strain loop analysis, can provide unique insights into these patients. In a recent study comparing 46 patients (eight women) with cardiac involvement and a preserved LVEF and diagnosed with either ATTR or HCM, Global Wall Impairment (GWI) was abnormally high in 93% of patients with ATTR-CA, particularly those in advanced NYHA functional classes II–IV, whereas it was increased in 43% of patients with HCM. Interestingly, women with ATTR-CA have lower absolute values of interventricular septum (IVS) thickness and left ventricular (LV) mass than their male counterparts, yet display a similar or even greater degree of functional impairment, as previously described. Thus, an assessment of myocardial work represents a potentially useful, sex-neutral parameter for determining disease severity in these women, who are typically incorrectly classified as having ‘low cardiac involvement’ by means of traditional echocardiographic parameters. By combining data from a myocardial work assessment with other parameters of functional impairment, including parameters from cardiopulmonary exercise testing and biomarker staging, it is therefore possible to develop and use a sex-adapted, comprehensive diagnostic framework for the early diagnosis and optimal individualized treatment of patients with ATTR before the onset of irreversible organ damage [38].

#### 3.3.3. Advanced Imaging: CMR, PET, and Nuclear Scintigraphy

Cardiac magnetic resonance imaging (CMR) can diagnose cardiac amyloidosis without the use of gadolinium-based contrast media in the majority of patients. Native myocardial T1 is markedly elevated in both AL and in most cases of late transthyretin-associated (ATTR) cardiac amyloidosis and accurately diagnoses suspected cases [39]. In a cohort of 868 patients being investigated for suspected cardiac amyloidosis at the Royal Free London NHS Trust, native myocardial T1 had a diagnostic area under the curve of 0.93. A simple diagnostic algorithm is proposed. A native T1 < 1036 ms (98% negative predictive value) or >1164 ms (98% positive predictive value) can be used to exclude or confirm the diagnosis of cardiac amyloidosis, with the intermediate range (1036–1164 ms) requiring further investigation, including contrast-enhanced CMR [40].

In a multicenter study of 160 patients with left ventricular hypertrophy of unknown cause at the University Clinic Freiburg, Germany, late gadolinium enhancement (LGE) had a sensitivity of 95% and specificity of 98% for the diagnosis of cardiac amyloidosis [41].

99mTc-pyrophosphate (PYP) scintigraphy has sex-related variability in uptake [42,43]. This variability was shown in a multi-institutional setting by studying 191 patients with no prior uptake on 99mTc-PYP scans. The mean and median bone pool activity counts, as well as the SUVmean were significantly higher in females compared to males (all *p* < 0.05) [35]. In a single-center retrospective review of 753 patients who underwent 99mTc-PYP imaging from 2010 to 2019, 41% (*n* = 307) had visual scores of 0, 23% (*n* = 177) had scores of 1 and 36% (*n* = 269) had scores of 2 and 3. Among 103 patients with a cardiac biopsy as the reference standard diagnosis, grade 2 and 3 99mTc-PYP scintigraphy had a 94% sensitivity and an 89% specificity for the diagnosis of ATTR [37].

### 3.4. Advanced Diagnostic Modalities and Sex-Specific Considerations

#### 3.4.1. Strain Imaging and Advanced Echocardiographic Parameters

In the largest prospective cohort of patients with symptoms of cardiac amyloidosis described to date, consisting of 1240 patients, including 766 with wtATTR and 474 with hATTR (follow-up 2000–2019), multiple echocardiographic parameters predicted patient death. These included stroke volume index, right atrial area index, longitudinal strain and the E/e’ ratio (all *p* < 0.05). Co-existent severe aortic stenosis predicted worse survival in patients with ATTR amyloidosis [44,45,46]. In a recent cohort of 82 patients with Val122Ile-related ATTR, women showed less negative global longitudinal strain (−10.6% ± 9.3% vs. −9.7% ± 10.0%, *p* = 0.003), potentially leading to delayed diagnosis in women if normal values for GLS are used without gender stratification [30].

#### 3.4.2. Cardiac Magnetic Resonance

Gadolinium-free cardiac amyloidosis diagnosis using CMR native T1 mapping is particularly useful in patients with renal dysfunction. The study evaluated the diagnostic value of native T1 mapping in 868 patients with suspected cardiac amyloidosis. A final diagnosis of cardiac amyloidosis was established in 436 patients (222 with AL and 214 with ATTR) and no cardiac involvement was found in 427 patients. Native T1 values were increased in both amyloid types. A simple clinical algorithm was proposed for the gadolinium contrast-free diagnosis of cardiac amyloidosis based on native T1 values. Native T1 values < 1036 ms had a 98% negative predictive value for cardiac amyloidosis, whereas T1 values > 1164 ms had a 98% positive predictive value for cardiac amyloidosis. Thus, native T1 values ≤ 1036 ms and ≥1164 ms can be used to rule in or rule out a diagnosis of cardiac amyloidosis, respectively. Patients with intermediate values (1036–1164 ms) could be spared from unnecessary gadolinium contrast administration, although only 58% of patients would fall into this intermediate range [40].

Myhir and all showed that intrinsic sex-based differences in myocardial tissue composition exist and are manifested by higher native T1 values in females. Higher mean native T1 values were found in females compared to males (1022 ± 23 ms vs. 1000 ± 22 ms, respectively, with a between-group difference of 22 ms at 95% CI). In a multivariable linear regression model including age, heart rate, left ventricular mass index, and blood T1, female sex was an independent predictor of native T1 (β 10 ms 95% CI). There was no interaction between sex and age (*p* = 0.27) [47].

Since the heart tissue of women is generally characterized by higher native T1 values than that of men, sex-specific T1 reference values have to be determined to allow for an accurate assessment of the affected heart tissue of patients with cardiac amyloidosis and for a precise prognosis of affected patients.

Standard diagnostic thresholds for T1 mapping have to be adapted for the baseline differences between the sexes.

Sex-adapted algorithms for the diagnosis of cardiac amyloidosis may allow to avoid overdiagnosis of amyloidosis in women as well as a delayed diagnosis due to the misinterpretation of higher T1 values as an exclusion criterion for amyloidosis.

Moreover, there are important sex differences in myocardial composition that have to be taken into account when calculating ECV. In a recently published cross-sectional study including 525 patients with a structurally normal heart, females had higher ECV values than males (26.5 ± 4.3% vs. 23.2 ± 3.7%, *p* < 0.001). Also, in a multivariable model, the sex differences persisted (β = 1.29, *p* = 0.02). There are also sex-specific optimal ECV cut-point values for mortality (31.3% for females and 27.5% for males, both *p* < 0.05/3) and thus sex-specific thresholds warrant the use of sex-adapted diagnostic and prognostic thresholds for ECV [48].

#### 3.4.3. PET Imaging

Published studies have described the excellent diagnostic capability of 18F-florbetaben PET/CT for the diagnosis of AL cardiac amyloidosis. Patients with AL amyloidosis have high, persistent uptake in the cardiac tissue that is sustained throughout the static imaging acquisition and is in marked contrast to the findings in ATTR and non-amyloid controls.

18F-Florbetapir positron emission tomography (18F-Florbetapir PET) showed different uptake patterns of 18F-Florbetapir in AL and in ATTR amyloidosis, allowing amyloidosis subtypes to be distinguished. In a dual-center, prospective study, 40 patients with cardiac amyloidosis proven by a biopsy and 20 non-amyloidosis patients and healthy controls were studied with 18F-Florbetapir PET/CT. All 20 patients with AL amyloidosis demonstrated intense early uptake in the heart with persistent high uptake at delayed frames. In contrast, 12 of 20 patients with ATTR amyloidosis showed early cardiac uptake that cleared rapidly. Mean standardized uptake values for AL were higher than those of ATTR at both early frames (5.55 vs. 2.55; *p* < 0.001) and delayed frames (3.50 vs. 1.25; *p* < 0.001). The use of 18F-Florbetapir PET/CT for cardiac amyloidosis imaging allowed accurate discrimination between AL and ATTR amyloidosis (100% accurate), although larger series will be needed to establish clinical usefulness [49].

Although several studies have quantified the amyloid burden and assessed the prognostic value of this imaging in patients with cardiac amyloidosis, no data on sex differences were found.

#### 3.4.4. Sex Differences in Cardiac Biopsy and Amyloidosis

Endomyocardial biopsy (EMB) is the gold standard for the definitive diagnosis and typing of cardiac amyloidosis. Women with ATTR-CM reach biopsy much later than men, with a median delay of 750 days compared to 86 days in men.

Despite available clinical guidelines for the diagnosis of cardiac amyloidosis, only 8.7% of women with ATTR-CM were diagnosed in the early disease phases, as opposed to 19.2% of men. The reasons for this significant diagnostic gap are the echocardiographic reference values for wall thickness, which are naturally lower for women than for men; thus, only women with very thick walls are currently considered for a biopsy diagnosis of cardiac amyloidosis. The endomyocardial biopsy itself does not have any documented sex-specific aspects, i.e., there are no technical differences and no sex-specific complications. However, women are underrepresented in reaching a biopsy diagnosis because of a delayed clinical suspicion [50].

### 3.5. Cardiac Biomarkers in Women

A recent German cohort analysis on cardiac amyloidosis underlines the differences in biomarkers of cardiac damage in women and men with amyloidosis. Patients with hereditary transthyretin amyloidosis showed significantly lower high-sensitivity troponin I (hs-cTnI) levels in women compared to men (women 30 ng/L vs. men 45 ng/L; *p* = 0.049) [1].

Biomarkers of cardiac damage showed sex-specific differences in patients with amyloidosis. Notably, troponin levels in the serum showed a correlation with the severity of 99mTc-PYP scintigraphy grades. The highest levels were observed in patients with grade 3 amyloidosis. A quantitative analysis of the PYP ratio on serum autoradiographs correlated significantly with the left ventricular mass and the interventricular septal thickness. Thus, the diagnostics of patients with cardiac amyloidosis can be optimized by using both biomarkers and imaging [51,52].

### 3.6. Sex-Specific Treatment Response and Outcomes

#### 3.6.1. ATTR Stabilizer Therapy: Tafamidis and Sex Differences

The transthyretin stabilizer tafamidis has received FDA approval and has become the standard of care for patients with ATTR [53]. In a new French Cardiac Amyloidosis Reference Center analysis of 1062 wild-type ATTR amyloidosis patients, important sex-specific differences in treatment efficacy were observed, with tafamidis associated with improved survival in men (adjusted hazard ratio 0.753, *p* < 0.001) but not in women. The reasons for these differences are unknown and could be due to a differing disease stage or phenotype at the time of treatment initiation [2]. In contrast, in patients with ATTRv Val122Ile amyloidosis, women treated with tafamidis showed a significantly better 48-month survival (0.452 vs. 0.767 hazard ratio reduction, *p* = 0.02) and a trend toward longer cardiac and global survival [30]. In a Spanish registry of 385 patients with all forms of ATTR amyloidosis, women received SGLT2 inhibitors significantly less frequently (15.8% vs. 27.2%, *p* = 0.024) and tafamidis (15.8% vs. 26.6%, *p* = 0.033) compared with men. These findings indicate that women with ATTR amyloidosis are undertreated [15].

These studies further support the need for a sex-adapted diagnostic and therapeutic strategy for cardiac amyloidosis. In particular, a sex-specific approach to the diagnosis and to the timing of treatment with TTR stabilizers could be translated into sex-specific diagnostic algorithms and criteria for the initiation of treatment. Future prospective studies of tafamidis in patients with ATTRwt should be characterized by comprehensive phenotyping in a sex-stratified manner. In this context, information on the extent of the disease (i.e., disease duration, amyloid burden) and on the degree of impairment of myocardial work and of the pressure–strain loop would be very valuable to identify patients with severe functional impairment and a preserved LVEF. Biomarker-guided disease staging, using both cardiac biomarkers (troponin and NT-proBNP) and markers of mitochondrial stress, would also be of great interest. These endpoints and the corresponding risk stratification algorithms should take into account the so-called “preserved-LVEF paradox” and the need for earlier treatment with tafamidis in order to preserve the remaining myocardial reserve in advanced stages of the disease in women with ATTR. Whether sex-specific adjunctive pathways—such as estrogen receptor modulation in postmenopausal women or mitochondrial-targeted antioxidant strategies—could influence the differential efficacy of TTR stabilizers remains speculative and requires prospective clinical validation [26].

#### 3.6.2. Treatment: Daratumumab-Based Regimens

Within the ANDROMEDA trial, 53.3% of 388 randomized patients with newly diagnosed AL amyloidosis treated with the daratumumab-containing regimen of DARA-VCd (daratumumab plus bortezomib, cyclophosphamide, and dexamethasone) achieved a hematologic complete response compared with 18.1% of patients treated with chemotherapy (*p* < 0.001). A cardiac response at 6 months was observed in 41.5% of patients treated with the daratumumab-containing regimens (renal response 53.0%) versus 22.2% of patients treated with chemotherapy [3]. Retrospective analyses within the pretreated AL amyloidosis population have also been summarized within a systematic review and meta-analysis that included 482 patients with AL amyloidosis treated with daratumumab-based regimens. The overall response rate to daratumumab monotherapy was 76% (95% CI, 68–84%), with a complete response rate of 30% and a very good partial response rate of 41%. The ANDROMEDA trial provides level 1 evidence that daratumumab combined with bortezomib, cyclophosphamide, and dexamethasone is the new standard of care for newly diagnosed AL amyloidosis, with 53.3% of 388 patients treated achieving a hematologic complete response compared with 18.1% while on chemotherapy (*p* < 0.001). In addition, daratumumab plus dexamethasone achieved an 81% overall response rate with a 51% complete response rate, and daratumumab-containing combination regimens achieved a greater than 80% response rate [54,55].

Data regarding sex-specific treatment responses within the ANDROMEDA trial are not available.

#### 3.6.3. Immunomodulator-Based Therapy in AL Amyloidosis

Immunomodulatory agents have emerged as important salvage therapies for relapsed AL amyloidosis. In a pooled analysis of three Phase II randomized trials involving 101 patients with light-chain amyloidosis treated with lenalidomide–dexamethasone, cyclophosphamide–lenalidomide–dexamethasone, or pomalidomide–dexamethasone, the median overall survival was 31 months and the median progression-free survival was 15 months; patients with a relapse-free very good partial response or better had a median relapse-free survival of 39 months [56]. In 40 patients with a relapse after the initial treatment for AL amyloidosis treated with the combination of the oral proteasome inhibitor ixazomib plus Iixazomib + lenalidomide + dexamethasone, 20.5% achieved a complete response, 20.5% a very good partial response and 17.9% a partial response [57].

Data regarding sex-specific treatment responses are insufficient.

#### 3.6.4. Treatment Strategies for Aortic Stenosis

Aortic stenosis (AS) is an increasingly recognized complication in the setting of cardiac amyloidosis, particularly in the context of wild-type transthyretin amyloidosis.

The prevalence of AS in wtATTR cardiac amyloidosis is particularly high, affecting up to 26% of patients with this form of amyloidosis. Importantly, this dual pathology carries a particularly poor prognosis with a higher mortality ranging from 24.5% to 60% at a long-term follow-up. Aortic valve replacement, performed via either transcatheter (TAVR) or surgical approaches, serves a critical life-saving function in amyloidosis patients who develop concomitant aortic stenosis. The primary purpose is to improve survival and reduce heart failure complications in this high-risk population. Notably, among the patients with AS and CA referred for TAVR, there is a marked male predominance (approximately 80% men) which may indicate underdiagnosis or under-recognition in women [58].

## 4. Conclusions and Future Directions

### 4.1. Summary of Key Findings on Sex-Specific Disparities

There is a growing recognition of the need for better awareness of the diagnostic and clinical features of women with amyloidosis. A number of observations have emerged as more cases have been evaluated and better diagnostic tools and therapies have been established. Women with amyloidosis are diagnosed at an older age with milder clinical features of cardiac involvement but with a greater impairment of functional status [1].

**Figure 2:**
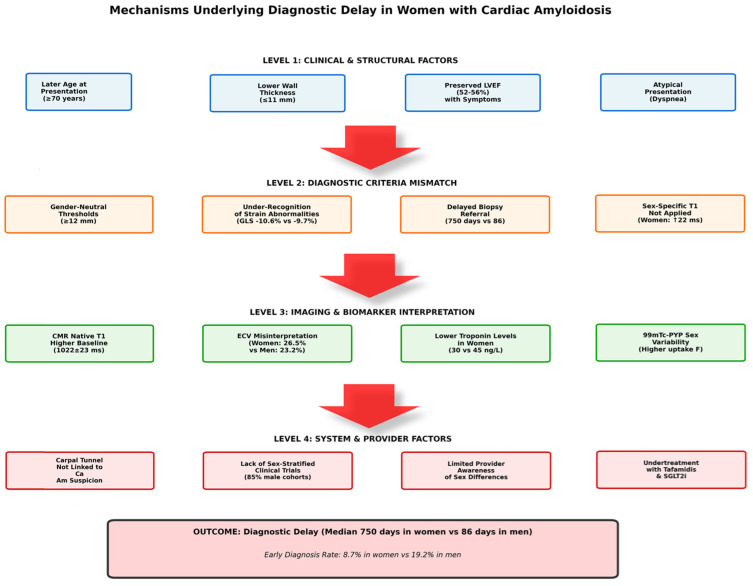
Mechanisms underlying the diagnostic delay for women with cardiac amyloidosis.

Mechanisms underlying the diagnostic delay for women with cardiac amyloidosis are summarized in Figure 2.

The use of sex-neutral diagnostic thresholds has been shown to result in underdiagnosis of women with amyloidosis, particularly among young women [2,6,7,10,15,30,53].

Women with cardiac amyloidosis experience a delay in diagnosis and limited access to treatment. In addition, the degree of treatment benefit for ATTR may vary between the sexes, while for patients with AL amyloidosis, there is lack of information regarding the efficacy of different therapeutic regimens in women [19].

The main sex-specific differences in cardiac parameters and clinical features are summarized in Table 1.

### 4.2. Recommendations for Clinical Practice

Heterogeneity in sex-based disparities in amyloidosis needs to be addressed at both clinical and research levels. A sex-based approach to diagnosing amyloid heart disease could begin with abandoning universal criteria such as wall thickness of ≥12 mm and instead using indexed measurements or sex-specific cut-points (≤11 mm in women vs. ≤12 mm in men) that are based on normative echocardiographic reference ranges [23,31]. Moreover, a multiparametric imaging strategy for detecting amyloidosis could incorporate established parameters (such as the left atrial strain, with a high sensitivity at a very good specificity (0.89 at 95% CI 0.82–0.94) and negative predictive value) and novel parameters (such as native T1 mapping for CMR, nuclear scintigraphy, in addition to wall thickness [35]. Importantly, the current clinical trials in amyloidosis have enrolled a predominantly male population (85% in the cohorts from the large randomized, placebo-controlled, genetic-diagnosis-blinded ATTR-ACT trials), and increasing the percentage of women enrolled in amyloidosis clinical trials is crucial to determine sex-specific treatment efficacy and to support the development of evidence-based, sex-adapted therapies [11,20].

Sex differences reported in some of the main recently published articles between women and men regarding the epidemiology, clinical features, diagnosis, and treatment of amyloid heart disease are summarized in Table 2.

### 4.3. Priority Research Questions for 2026 and Beyond

There are many areas where knowledge gaps need to be addressed by future prospective studies: (1) Mechanistic studies as to why women with ATTR have a preserved LVEF while being more ill and having more extracardiac symptoms than men, whether there is a role for hormonal studies to better understand gender differences in heart disease, and the baseline physiological differences [1]. (2) Validation of the proposed sex-specific diagnostic strategies for the use of indexed wall thickness, strain imaging, and biomarkers to facilitate an earlier diagnosis of cardiac amyloid disease in women [23]. (3) The design of sex-stratified, randomized controlled trials of therapies that stabilize, reduce the production of, or silence transhyretin (ATTR) and supportive therapies, in which efficacy may or may not differ among women [2,30]. (4) An assessment of the barriers and strategies to increase female enrollment in AL amyloidosis clinical trials and methods to achieve a sex balance in clinical trials so that they reflect the sex-based prevalence of the disease [12].

Without such interventions, women with cardiac amyloidosis will continue to face diagnostic delays and treatment uncertainties rooted not in biological differences but in a research methodology biased toward male representation.

## Figures and Tables

**Table 1:** Sex-specific differences in cardiac parameters and clinical features [1,2,13,15,18,20,23,24,30,31,32,33,35,50,51].

Clinical Parameter	ATTR (Males)	ATTR (Females)	Al (Males)	AL (Females)
LV Wall Thickness	Higher	Lower	Variable	Variable
LVEF	Lower (45–50%)	Higher (50–55%)	Variable	Variable
NT-proBNP	More elevated	Less elevated	More elevated	Variable
Troponin	More elevated	Less elevated	More elevated	Variable
Carpal Tunnel Syndrome	40–50%	50–60%	Rare (<5%)	Rare (<5%)
Neuropathy	Less common	More common	Common	Common
Age at Diagnosis	70–85 years	75–90 years	60–65 years	60–68 years
Disease Severity at Dx	Severe	Milder	Variable	More systemic
Tafamidis Benefit	Clear benefit	Limited benefit	N/A	N/A

**Table 2:** Sex differences reported in some of the main recently published articles between women and men regarding the epidemiology, clinical features, diagnosis, and treatment of amyloid heart disease.

Study Reference	Publication Year	Epidemiology/Demographics	Clinical Features	Diagnosis	Treatment/Outcomes
Vogel et al. [1]	2025	Female:male ratio ~1:7 (14.2% women, 86% male). Women diagnosed significantly later (median 750 vs. 86 days, *p* = 0.022). Median age 82 years for both.	Women: lower interventricular septal diameter, lower LV mass, higher ejection fraction. Women: lower troponin I levels, worse renal function. Functional capacity remained lower in women despite comparable NYHA class.	Diagnostic delay significantly longer in women. Lower IVS thickness in women may mask disease using standard ≥12 mm criteria. Sex-specific echocardiographic differences persisted after 6–12 months therapy.	Tafamidis therapy: sex-specific differences persist. Echocardiographic parameters remain different between sexes.
Zaroui et al. (ATTRwt) [2]	2025	1062 ATTRwt-CM patients (180 women, 16%). Women significantly older at diagnosis (median LVEF 52% vs. 50% in men). Younger women (≤77 years) had higher death rate.	12% of women vs. 4.1% of men had septum <12 mm (*p* = 0.004). Women: higher LVEF, lower interventricular septum thickness, better global longitudinal strain. Younger women: higher risk of sudden death (13.8% vs. 4.6%; OR 3.24; *p* = 0.001).	Women with thin LV walls but high death rate suggests diagnostic miss. Septum thickness cutoff <12 mm would increase ATTR-CM diagnosis frequency in women. Sex-age interaction in diagnostic presentation.	Tafamidis: protective in men (HR 0.61) but not in women. Younger women have distinct phenotype requiring sex-adapted diagnostic criteria.
THAOS Analysis (Mora-Ayestarán et al.) [13]	2024	1251 ATTRwt amyloidosis patients. Female proportion increased with age: 4.1% (<70 years) to 14.3% (≥90 years). Greatest diagnostic delay in women <70 years (5.2 vs. 1.3 years in men).	Karnofsky Performance Status ≤70: 17.1% (<70 yrs), 30.1% (70–79 yrs), 46.1% (80–89 yrs), 44.4% (≥90 yrs).	Diagnostic delay: overall 2 years from symptom onset; women <70 years: 5.2 years. Significant diagnostic challenges in younger women.	Sex-specific diagnostic algorithms recommended.
Enríquez-Vázquez et al. (AMIGAL Registry) [15]	2026	385 ATTR-CA patients: 95 women (24.7%), 290 men (75.3%). Median age 82.5 years.	Women: more frequently NYHA class ≥III (36.8% vs. 25.2%, *p* = 0.028). Women: higher LVEF (56.0% vs. 52.6%, *p* = 0.003), higher indexed LV max thickness (10.2 vs. 9.2 mm/m^2^, *p* = 0.001).	No differences noted.	Women: less HF hospitalization (IR 167.39 vs. 245.61, *p* = 0.033). Women received less SGLT2i (15.8% vs. 27.2%, *p* = 0.024) and tafamidis (15.8% vs. 26.6%, *p* = 0.033). Similar survival (4.1 years both sexes).
Aimo et al. (Sex-Specific Cut-offs) [24]	2023	302 ATTR-CA patients: 49 women (16%), older than men (83 vs. 80 years, *p* = 0.009). Similar survival and HF outcomes.	Women: lower IVS (15 vs. 17 mm), lower posterior wall thickness (13 vs. 15 mm). When indexed by height/BSA: differences disappeared. Equal relative wall thickness (0.61 vs. 0.69; *p* = 0.448).	Current ≥12 mm cutoff may cause diagnostic delay in women. Index measures similar; suggest sex-specific cutoffs (11 mm women vs. 12 mm men) or height indexing.	Similar NYHA class, NT-proBNP, comorbidities, outcomes. Indexed or sex-specific criteria recommended.
Zaroui et al. (Clustering Analysis) [20]	2025	2233 patients (1659 men, 574 women). AL amyloidosis more prevalent in women, especially in systemic involvement clusters. ATTRwt dominated in men (Clusters A, B, C).	Men: higher comorbidities (hypertension, diabetes), more severe cardiac involvement. Women: better outcomes in advanced disease (Cluster D) vs. men. Similar mortality patterns by cluster (~23–25.8% at 5 years).	Distinct sex-specific phenotypes highlighted by clustering. Men had worse survival in advanced disease; women had higher mortality despite milder cardiac features in mid-life clusters.	42.5% of all patients experienced death or heart transplantation at 5 years. Sex-specific risk stratification and treatment strategies needed.
Vogel et al. (Gender Diagnostic Gap) [50]	2025	240 ATTR-CM: 34 (14.2%) women. Fewer women diagnosed in early period (8.7% vs. 19.2%, *p* = 0.019). Shorter diagnostic time in later period for women.	Women: thinner IVS (15.9 ± 4.5 vs. 18.1 ± 4.5 mm, *p* = 0.026), lower stroke volume, higher LVEF (55% vs. 51.7%, *p* < 0.001), lower LV mass. Lower troponin I (30 vs. 45 ng/L, *p* = 0.049).	Current ≥12 mm thresholds don’t adequately reflect female patterns. Diagnostic period affected detection rate. Sex-adapted diagnostic criteria essential.	Echocardiographic differences persisted after 6 months stabilizer therapy.
Patel et al. (ATTR-CM Phenotypes) [18]	2022	1732 ATTR patients studied: wtATTR-CM 6% women; T60A-hATTR-CM 29.6% women; V122I-hATTR-CM 27.8% women. Women 3.3 years older at diagnosis across types.	When indexed, structural/functional phenotype similar between sexes. Non-indexed measures suggested mildly worse disease in females. Body size significantly influenced severity measures.	Non-indexed wall thickness measurements contributed to under-representation and diagnostic delays in women. Sex-adapted, indexed echocardiographic parameters recommended.	No significant sex differences in progression, mortality across population (*p* = 0.459). Similar prognosis when properly indexed.

## Data Availability

No new data were created or analyzed in this study.

## Abbreviations

The following abbreviations are used in this manuscript:

18F-Florbetapir PET	18F florbetapir positron emission tomography
AA	serum amyloid A amyloidosis
AL	light chain amyloidosis
AS	aortic stenosis
ATTR	transthyretin amyloidosis
hATTR	hereditary transthyretin amyloidosis
vATTR	variant transthyretin amyloidosis
wtATTR	wild-type transthyretin amyloidosis
ATTR-CM	transthyretin amyloidosis cardiomyopathy
CPET	cardiopulmonary exercise testing
CMR	cardiac magnetic resonance imaging
Cts	carpal tunnel syndrome
ECV	extracellular volume
EMB	endomyocardial biopsy
FLC-MS	measurement of free light chain mass
hs-cTnI	high-sensitivity troponin I
LGE	late-gadolinium-enhancement
LV	left ventricular
LVEF	left ventricular ejection fraction
NYHA	New York Heart Association
TAVR	transcatheter aortic valve replacement
TTR	transthyretin
6MWT	6 min walking test
